# Auditory–Olfactory Integration in Simulated Natural Environments Dissociates Neural and Subjective Responses in Visually Impaired Individuals

**DOI:** 10.3390/biology15191705

**Published:** 2026-09-24

**Authors:** Xiuli Xie, Jingyu Wu, Yiwei Zhou, Hua Chen

**Affiliations:** 1 School of Life Sciences, Zhaoqing University, Zhaoqing 526061, China; 2 Guangdong Provincial Key Laboratory of Ornamental Plant Germplasm Innovation and Utilization, Environmental Horticulture Research Institute, Guangdong Academy of Agricultural Sciences, Guangzhou 510640, China; 3 Faculty of Innovation and Design, City University of Macau, Macau 999078, China

**Keywords:** visual impairment, multisensory integration, EEG, α/β ratio, cross-modal plasticity, olfactory–auditory interaction, environmental neuroscience

## Abstract

Visually impaired individuals rely on cross-modal plasticity to compensate for absent visual input, yet the neurophysiological mechanisms underlying naturalistic auditory–olfactory integration remain poorly characterized. This study investigated how combined auditory and olfactory cues in representative landscape scenarios modulate cortical activity and multidimensional perceptual experience in 20 visually impaired participants. Five ecologically distinct scenarios—wooded resting areas, dynamic water features, aromatic planting beds, open lawns, and hard-paved plazas—were simulated using synchronized playback of field-recorded soundscapes and calibrated odor diffusion systems. Participants completed standardized ratings across auditory, olfactory, and integrated perceptual dimensions while electroencephalographic (EEG) recordings were acquired. Occipital α/β ratios (O1/O2) served as the primary neurophysiological index of cortical relaxation. Results demonstrated a dissociation between explicit hedonic appraisal and implicit neurophysiological response: dynamic water soundscapes dominated subjective preference ratings, whereas floral olfactory cues in low-amplitude acoustic contexts elicited the highest α/β ratios, significantly exceeding those recorded in wooded and open lawn scenarios. Odor familiarity correlated inversely with α/β elevation (r = −0.46, *p* < 0.05), indicating that unfamiliar floral scents triggered more robust relaxation responses than familiar urban olfactory environments. This correlation may reflect novelty-related attentional processes rather than relaxation per se. Exploratory clustering revealed stable, trait-like individual differences in baseline α/β activity that were not explained by perceptual ratings, and linear mixed-effects modeling confirmed that inter-individual baseline differences, rather than linear coupling with conscious evaluations, drove α/β variance. Given the relatively small sample size, these findings should be regarded as preliminary and hypothesis-generating. Nevertheless, they extend current models of cross-modal neuroplasticity by demonstrating that naturalistic multisensory integration follows dissociable subjective and neurophysiological trajectories, informing evidence-based multisensory interventions for accessible environmental design.

## Simple Summary

People who are blind or have very low vision rely on their other senses—especially hearing and smell—to understand and move through the world around them. However, most public parks and outdoor spaces are designed mainly for sighted visitors, with little thought given to how they feel, sound, or smell to someone who cannot see. This study set out to understand how different combinations of natural sounds and odors affect both the emotional experience and brain activity of visually impaired people. Twenty volunteers with visual impairments took part in a laboratory experiment where they were exposed to five different simulated park environments, each pairing realistic nature sounds with matching pleasant odors. While listening and smelling, participants rated how much they liked each setting, and their brain activity was measured using a lightweight, wireless EEG recorder with electrodes placed on the scalp. The results showed something surprising: the environments that people said they liked best—those with the sound of flowing water—did not actually produce the strongest signs of relaxation in the brain. Instead, quiet spaces with floral scents triggered the deepest relaxation responses, even though people did not always rate them as their favorites. The study also found that each person’s brain responded differently, suggesting there is no one-size-fits-all solution for accessible design. However, because the number of participants was relatively small, these findings should be considered preliminary and will need to be confirmed in larger studies. These findings can help architects, city planners, and rehabilitation specialists create parks and public spaces that do not just look good, but actually help visually impaired people feel calm, comfortable, and more connected to their surroundings. By combining scientific brain measurements with real-world design, this work points toward a future where outdoor spaces are truly welcoming to everyone, regardless of how they see the world.

## 1. Introduction

According to the World Health Organization, at least 2.2 billion people worldwide experience some degree of visual impairment, of whom at least 1 billion have an impairment that could have been prevented or is yet to be addressed [1]; the Global Burden of Disease study estimated 43.3 million people were blind in 2020 [2]. For individuals with visual impairments, independent and safe mobility in outdoor environments constitutes a fundamental prerequisite for social participation and self-realization. Central to this capability is the effective perception and interpretation of surrounding environments. The environmental perception needs of visually impaired populations extend beyond mere physical accessibility; they encompass unimpeded multimodal perception, whereby individuals construct holistic, stable, and meaningful environmental imagery through multimodal sensory channels [3,4]. Existing studies indicate that visually impaired individuals rely on non-visual senses to acquire spatial information for wayfinding. Environmental architectural elements—including material transitions, topographic gradients, component arrangements, and acoustically reflective spatial configurations—provide functional cues across both two-dimensional planes and three-dimensional volumes [5], thereby establishing a foundation for understanding non-visual perceptual patterns and informing evidence-based design principles. However, most of this evidence derives from controlled laboratory settings or individual sensory modalities. How these perceptual patterns operate within naturalistic multisensory environments remains less understood.

Nevertheless, contemporary urban public space and landscape design remain predominantly vision-centric, prioritizing spatial form, visual aesthetics, and scenic composition while affording limited consideration to the non-visual sensory experiences—a tendency widely characterized as “ocularcentrism.” In response, scholarship has increasingly explored how outdoor environments can redress this bias through multisensory design pathways, exemplified by sensory gardens that promote outdoor well-being among visually impaired populations [6].

Owing to constrained access to visual information, visually impaired individuals depend more heavily on auditory, olfactory, and tactile modalities to support spatial cognition, environmental comprehension, and behavioral decision-making. Consequently, they encounter pronounced challenges in environmental perception, spatial orientation, and emotional experience within traditionally vision-dominated public spaces [7,8]. Research on blind pedestrians’ wayfinding strategies across varied urban sensory contexts reveals that in dense urban areas, ambient soundscapes and environmental echoes serve as primary navigational cues, with enclosed boundaries facilitating orientation. By contrast, park environments—characterized by diffuse edges and reduced enclosure—exhibit altered sensory hierarchies [8]. This shift underscores the significance of landscape settings as a key variable in perceptual research: the inherent ambiguity of spatial boundaries and the concurrent layering of multisensory cues in garden landscapes warrant finer-grained investigation. Grounded in the principle of sensory compensation, spatial environmental information is acquired through the coordinated action of vision, audition, touch, olfaction, and their multimodal integration. When one sensory modality is diminished or absent, the sensitivity of remaining modalities heightens to compensate—a phenomenon extensively corroborated by cross-modal neuroplasticity research concerning auditory, tactile, and olfactory channels in visually impaired populations [9,10,11,12]. These studies have predominantly examined each sensory channel in isolation. The interactive effects of simultaneous auditory and olfactory input on cortical reorganization remain largely unexplored.

In the tactile domain, tactile paving and raised-relief maps supply localized, precise path information; however, their limited coverage, susceptibility to damage, and environmental interference constrain practical utility. Wearable electronic travel aids, despite ongoing technological refinement, similarly face challenges related to ergonomic burden, environmental adaptability, and operational reliability [13]. In the auditory domain, natural sounds, traffic flows, ambient noise, and electronic navigation prompts furnish rich dynamic cues, enabling visually impaired individuals to localize themselves and navigate via acoustic landmarks [13]. Yet auditory information is inherently transient and directional, rendering it less effective in noisy environments or static spaces lacking distinctive sound sources [14]. Moreover, natural soundscapes have been shown to mitigate anthropogenic noise stress and facilitate psychological restoration, highlighting the potential affective value of acoustic environmental quality for visually impaired users [15]. Auditory perception in visually impaired populations has thus received substantial research attention. By contrast, the role of olfaction in environmental experience has been examined far less extensively.

Despite this relative neglect, olfaction possesses a distinctive capacity for emotional arousal—the Proust effect—whereby odors evoke profound episodic memories and affective responses. This mechanism enables visually impaired individuals to construct personalized, emotionally resonant cognitive maps, strengthening spatial identification and place attachment [16]. Research on auditory environmental perception among visually impaired populations has matured considerably, encompassing echolocation, acoustic landmark recognition, soundscape preferences, and park acoustic environment evaluation [17,18]. By comparison, olfactory perception research has focused primarily on indoor safety, isolated odor identification, odor–memory associations [19], and enhanced olfactory discrimination among congenitally blind children and adolescents [20,21].

Crucially, in dynamic and complex real-world environments, audition and olfaction do not operate in isolation but interact synergistically in real time. This interdependence is particularly salient in landscape settings, where multisensory cues converge: auditory stimuli (e.g., rustling leaves, flowing water, birdsong) and olfactory cues (e.g., floral fragrances, vegetal aromas, earthy scents) co-occur to shape users’ environmental perception and affective experience [22]. For visually impaired individuals, who rely on non-visual modalities, such audio-olfactory interactions are pivotal to the integrity of spatial cognition and the richness of place experience [23]. EEG has been widely used to evaluate multisensory perception in sighted populations, revealing how concurrent sensory inputs modulate cortical dynamics [24]. Although emerging work has begun to construct parallel auditory–olfactory perception models for urban visually impaired populations from a sensory justice perspective [5] and exploratory case studies have examined multisensory engagement in accessible gardens through behavioral mapping [25], research on audio-olfactory interaction remains nascent. Empirical investigations linking auditory–olfactory interaction to subjective perception and electroencephalographic activity across diverse landscape scenarios are notably lacking. In particular, whether conscious perceptual evaluations align with implicit neurophysiological responses under simultaneous audio-olfactory exposure remains unknown.

Previous studies have examined auditory and olfactory perception separately, often with artificial stimuli in laboratory settings. The simultaneous presentation of both modalities within naturalistic landscape contexts has not been addressed. Accordingly, this study employed a controlled multisensory paradigm to investigate auditory–olfactory interactions in 20 visually impaired participants across five representative landscape scenarios. Subjective perception ratings and EEG recordings were collected simultaneously. This combination of subjective and neurophysiological measures distinguishes the present work from prior studies that relied on either self-reports or EEG alone. We examined whether subjective evaluations and cortical responses converge or diverge across different stimulus combinations. We expected scenario-specific patterns, with possible dissociations between conscious appraisal and implicit neural activity. The findings inform scenario-adaptive sensory optimization strategies for accessible design and contribute foundational neurophysiological evidence to the literature on non-visual spatial perception. The following sections describe the experimental methods, present the results, and discuss their implications for multisensory environmental design.

## 2. Materials and Methods

### 2.1. Experimental Scenarios

To investigate the influence of multisensory environmental features across different landscape settings on visually impaired individuals’ perceptual and affective experiences, this study selected representative park spatial typologies as experimental scenarios. Scenario design integrated three dimensions—spatial configuration, typical environmental sounds, and olfactory elements—to construct experimental conditions that approximated authentic environmental features (Table 1).

In accordance with the Chinese national standard Code for Park Design (GB 51192-2016 [26]), five typical landscape scenarios were identified: wooded resting areas, dynamic water features, aromatic planting beds, open lawns, and hard-paved plazas. For each scenario, characteristic auditory and olfactory elements were extracted (Table 1). Auditory stimuli were high-fidelity field recordings captured in corresponding real-world settings. The auditory stimuli are available as Audio S1 in the Supplementary Materials. Olfactory stimuli were formulated by Hangzhou Aroma Kingdom Technology Co., Ltd. (Hangzhou, China), based on specified target odor profiles, using customized essential-oil blends. Professional odor-evaluation technicians verified the fidelity of each formulation against its intended referent. Odor delivery was achieved via neck-worn digital aroma emitters.

### 2.2. Participants

Twenty visually impaired individuals (14 males, 6 females; aged 16–26 years) participated in the experiment, encompassing both low-vision and totally blind individuals (Table S1). The cohort comprised 14 participants with congenital visual impairment and 6 with acquired visual impairment. Detailed ophthalmological records (e.g., confirmed etiology, visual acuity measurements) were not available for all participants. All participants were free from major medical conditions, including neurological, psychiatric, or olfactory disorders. Pre-experimental screening confirmed that systolic blood pressure, diastolic blood pressure, and heart rate fell within normal clinical ranges. All participants self-reported normal hearing and no history of hearing disorders, and confirmed verbally that they could clearly hear the auditory stimuli during the familiarization phase. Participants also self-reported a normal sense of smell and no history of olfactory disorders, and were instructed to avoid using scented products on the day of the experiment. Written informed consent was obtained from all participants prior to testing, and confidentiality of personal information disclosed in questionnaires was strictly maintained.

### 2.3. Apparatus and System Configuration

The experimental setup simulated the five landscape scenarios within a controlled laboratory environment, synchronizing scenario-specific soundscapes with botanical olfactory cues. Neck-worn wearable aroma emitters delivered precisely timed olfactory stimuli, while a wireless physiological recording system acquired electroencephalographic (EEG) data in real time to objectively quantify cortical responses. Subjective evaluations—encompassing perceived safety, spatial legibility, affective preference, and overall comfort—were collected through structured questionnaires supplemented by semi-structured interviews.

#### 2.3.1. Soundscape Presentation System

Auditory stimuli consisted of high-fidelity field recordings. Recordings were made with a Sennheiser AMBEO binaural in-ear microphone set (Sennheiser electronic GmbH & Co. KG, Wedemark, Germany) connected to a Zoom F6 portable field recorder (Zoom Corporation, Tokyo, Japan) at 96 kHz/24-bit resolution. Files were edited in Adobe Audition (Adobe Inc., San Jose, CA, USA) with noise reduction and RMS normalization to −20 dBFS. The final audio files are provided as Supplementary Materials (Audio S1) to facilitate replication. During the experiment, audio was presented via a Lenovo Xiaoxin Pro 16 laptop (Lenovo, Beijing, China) running E-Prime 3.0 (Psychology Software Tools, Sharpsburg, PA, USA), routed through a Focusrite Scarlett 18i8 USB audio interface (Focusrite plc, High Wycombe, UK) to a pair of Yamaha HS5 active studio monitors (Yamaha Corporation, Hamamatsu, Japan). Sound pressure level at the participant’s ear position was calibrated to 65 dB(A) using a sound level meter before each session. Each scenario’s audio file was verified prior to the experiment to confirm correct playback and output level.

#### 2.3.2. Olfactory Delivery System

Odors were delivered using a neck-worn scent player (X-SCENT 3.0, Hangzhou Aroma Kingdom Technology Co., Ltd., Hangzhou, China). The device was worn around the neck, with the outlet positioned roughly 25 cm below the nose. Odor cartridges were prepared by the manufacturer based on our target profiles, using essential-oil blends. The specific formulations and concentrations were determined by the manufacturer to match the target olfactory profiles of each landscape scenario. Liquid odorants were released at a constant flow rate of 0.1 mL/s, and the release timing was controlled by the same script that triggered the audio files, so sound and smell started together. Each cartridge was used for one scenario only. Between scenarios, the room was ventilated for 5 min, and the device was purged with clean air before the next condition started. We did not independently measure absolute odor concentration in the breathing zone, nor did we formally confirm its spatial and temporal stability. To mitigate this limitation, we kept the same cartridge lots, release duration, and flow rate across all participants to maintain relative concentration consistency. Additionally, the 5 min ventilation and clean-air purge between scenarios were intended to minimize olfactory carryover and habituation effects.

#### 2.3.3. Physiological Signal Acquisition System

EEG data were acquired using a PhysioLAB wireless physiological recorder (ERGONEERS GmbH, Egling, Germany). Of the unit’s nine configurable sensor channels, three were allocated to EEG. Three active electrodes were placed according to the modified 10–20 system at O1, O2, and Cz, with linked mastoids (A1/A2) as reference and AFz as ground. The hardware sampling rate was 4000 Hz per channel, down-sampled to 1000 Hz for analysis. Impedance was kept below 10 kΩ at all sites. The present study focused on alpha (α), beta (β), and theta (θ) frequency bands to characterize changes in emotional valence and cognitive state across experimental conditions. For each scenario, the 2 min recording was segmented into 2 s epochs. Artifact-free epochs were retained and averaged to compute spectral power for each frequency band.

#### 2.3.4. Questionnaire Instrument

A structured questionnaire was developed to assess participants’ subjective evaluations of auditory, olfactory, and integrated auditory–olfactory perception. The instrument comprised three sections: (i) auditory perception, measuring acoustic comfort, pleasantness, and preference; (ii) olfactory perception, measuring odor comfort, familiarity, and preference; and (iii) integrated auditory–olfactory environmental perception, measuring spatial legibility, environmental comfort, and overall environmental preference (Table S2). Each item was rated on a 5-point Likert scale. The questionnaire was administered orally by a trained researcher immediately after each scenario exposure, with participants instructed to rate each modality separately before providing their integrated assessment.

### 2.4. Experimental Procedure

#### 2.4.1. Preparation Phase (~5 min)

Participants were seated in a comfortable armchair in the laboratory’s testing chamber. After skin preparation with alcohol wipes, EEG electrodes were applied and impedance checked to ensure all channels were below 10 kΩ. Participants were then fitted with the neck-worn aroma emitter. The experimental procedure was explained verbally (supplemented by large-print instructions for low-vision participants), written informed consent was obtained, and demographic/clinical information was collected via a brief intake form.

#### 2.4.2. Experimental Phase (50 min)

Five landscape scenarios—aromatic planting beds, wooded resting areas, dynamic water features, open lawns, and hard-paved plazas—were presented in a Latin-square counterbalanced order to control for sequence and carryover effects. Within each scenario, auditory and olfactory stimuli were delivered simultaneously. Each scenario was presented once per participant. Participants experienced each scenario for 2 min while maintaining a quiet, stationary posture with eyes closed (to eliminate residual light interference). Immediately following each exposure, participants completed the scenario-specific questionnaire via oral interview administered by a trained researcher, with responses recorded verbatim. Between consecutive scenarios, the laboratory underwent active forced-air ventilation for 5 min to minimize residual odorants and prevent sensory adaptation or cross-contamination. Additionally, a 30 s clean-air purge of the olfactory delivery system was performed between conditions to minimize carry-over effects. The procedure cycled through all five scenarios until completion.

### 2.5. Data Processing and Analysis

#### 2.5.1. EEG Signal Processing

Raw EEG signals were preprocessed to enhance data quality. Signals were sampled at 1000 Hz. A 50 Hz notch filter was applied to suppress power-line interference, followed by a 1–40 Hz band-pass filter to retain physiologically relevant frequency components. Continuous data were segmented into 2 s non-overlapping epochs. Artifact rejection was performed using an amplitude threshold of ±100 μV to minimize contamination from ocular movements and electromyographic activity. Data segments exhibiting excessive noise or signal dropout were excluded; 20 valid datasets remained for subsequent analysis. Spectral analysis employed Welch’s power spectral density estimation, applied within each 2 s epoch, with a 2 s Hamming window and 50% overlap, to derive frequency-domain features. Frequency bands were defined as follows: θ (4–8 Hz), α (8–13 Hz), and β (13–30 Hz). Alpha (α), beta (β), and theta (θ) power values were extracted, and the ratios α/β, θ/β, and (α + θ)/β were computed, serving as neurophysiological indices of emotional and cognitive modulation under varying environmental stimuli. The α/β ratio is a widely used index of cortical relaxation versus arousal [27,28,29]. All preprocessing and artifact rejection steps were performed using a fully automated, uniform pipeline applied identically across all experimental conditions. This standardized approach ensured consistent data quality across the five scenarios, with no condition requiring disproportionate data exclusion.

#### 2.5.2. Questionnaire Data Processing

Questionnaire responses were coded and compiled. Descriptive statistics—including means and standard deviations—were computed for each evaluative dimension to characterize overall trends in subjective ratings across landscape scenarios. Internal consistency reliability (Cronbach’s α) was assessed to validate the measurement instrument. Comparative analyses across scenarios established a foundation for inferential statistical testing.

#### 2.5.3. Statistical Analysis

All statistical analyses were conducted in R version 4.3.1. The analytical plan comprised prespecified (confirmatory) and exploratory components, as detailed below.

Prespecified analyses. Linear mixed-effects models (LMMs) served as the primary analytical approach. For each EEG ratio (α/β, θ/β, and (α + θ)/β), an LMM was fitted with scenario as a fixed effect, participant ID as a random intercept, and the corresponding ratio as the dependent variable. This approach accounted for the repeated-measures structure and high individual variability. Model assumptions were assessed using residual diagnostic plots, which indicated adequate adherence to normality and homoscedasticity assumptions. For questionnaire ratings, Friedman’s two-way ANOVA by ranks evaluated differences across the five scenarios, with post hoc pairwise comparisons performed using Wilcoxon signed-rank tests with Bonferroni correction. For EEG metrics, one-way repeated-measures ANOVA (RM-ANOVA) assessed between-scenario differences, with Mauchly’s test used to verify sphericity assumptions. When sphericity was violated, degrees of freedom were corrected using the Huynh–Feldt estimate. Post hoc pairwise comparisons following significant RM-ANOVA effects were conducted using paired *t*-tests with Bonferroni correction, based on the RM-ANOVA pooled error term. Prior to parametric analyses, normality was assessed using the Shapiro–Wilk test. Effect sizes were reported for all prespecified analyses: partial η^2^ for RM-ANOVA, Cohen’s d for pairwise comparisons, Kendall’s W for Friedman’s test, and marginal R^2^ for LMMs, each with 95% confidence intervals.

Exploratory analyses. Spearman rank correlation examined relationships between neurophysiological indices and subjective ratings, as well as between odor familiarity and EEG ratios; correlation coefficients (*r*) and corresponding *p*-values are reported. Cluster analysis was performed using K-means (with silhouette analysis determining optimal cluster count) and hierarchical cluster analysis (HCA) using Ward’s linkage method with Euclidean distance, to identify distinct neurophysiological response profiles. Given the exploratory nature of these analyses, *p*-values are interpreted with caution, and findings are considered hypothesis-generating rather than confirmatory. No formal correction for multiple comparisons was applied to the exploratory analyses; instead, effect sizes and *p*-values are provided to facilitate interpretation.

## 3. Results

### 3.1. Auditory Perception Ratings

Significant differences emerged across the five simulated scenarios for all three auditory perception dimensions (Figure 1). A Friedman test revealed a significant effect of scenario on sound comfort (χ^2^(4) = 18.43, *p* = 0.00102, Kendall’s W = 0.23); sound pleasantness (χ^2^(4) = 18.95, *p* = 0.000804, Kendall’s W = 0.24); and sound preference (χ^2^(4) = 23.88, *p* = 0.000084, Kendall’s W = 0.30). The Dynamic Water Feature Area (DWA) attained the highest mean scores on sound comfort, sound pleasantness, and sound preference. Post hoc pairwise comparisons revealed an identical pattern across dimensions: DWA scored significantly higher than the Aromatic Flowerbed Area (AFA), Open Lawn Area (OLA), and Hard-paved Activity Plaza Area (HPA) (all Z > 2.8, all *p* < 0.05 after Bonferroni correction), but did not differ significantly from the Shaded Rest Area (SRA). Additionally, SRA scored significantly higher than HPA on sound preference (Z = 2.85, *p* = 0.044), though no significant differences were detected between SRA and either AFA or OLA.

### 3.2. Olfactory Perception Ratings

Among the three olfactory perception dimensions, only odor familiarity exhibited significant variation across the five scenarios (χ^2^(4) = 11.42, *p* = 0.022, Kendall’s W = 0.14), whereas odor comfort (χ^2^(4) = 3.47, *p* = 0.482) and odor preference (χ^2^(4) = 2.57, *p* = 0.632) did not differ significantly (Figure 2). Odor familiarity peaked in the HPA scenario, with scores significantly exceeding those recorded in DWA (Z = 3.05). No significant differences were found between HPA and AFA, SRA, or OLA.

### 3.3. Integrated Multisensory Perception Ratings

All three integrated perception dimensions differed significantly across the five scenarios (Figure 3). A Friedman test revealed a significant effect of scenario on spatial legibility (χ^2^(4) = 18.19, *p* = 0.00113, Kendall’s W = 0.23), environmental comfort (χ^2^(4) = 10.82, *p* = 0.0287, Kendall’s W = 0.14), and environmental preference (χ^2^(4) = 15.88, *p* = 0.00318, Kendall’s W = 0.20). For spatial legibility, DWA and HPA achieved the highest mean scores, both significantly surpassing AFA (Z = 4.03 and 5.44, respectively) but not differing significantly from SRA or OLA. Regarding environmental comfort, DWA recorded the highest mean score, significantly exceeding HPA (Z = 2.95) but showing no significant differences relative to AFA, SRA, or OLA. For environmental preference, DWA again yielded the highest mean score, significantly outperforming AFA (Z = 4.31), OLA (Z = 3.89), and HPA (Z = 5.16), with the sole exception of no significant difference compared with SRA.

### 3.4. Electroencephalographic Findings

Repeated-measures ANOVA revealed a significant main effect of scenario on the α/β ratio (F(2.22, 42.24) = 3.31, *p* = 0.048, partial η^2^ = 0.15), but not on the θ/β ratio (F = 2.36, *p* = 0.093) or the (α + θ)/β ratio (F = 0.93, *p* = 0.376) (Figure 4). Post hoc pairwise comparisons with Bonferroni correction for the α/β ratio indicated that the AFA scenario elicited a significantly greater mean α/β ratio compared to SRA (t(76) = 3.15, *p* = 0.023, 95% CI [0.04, 0.90]) and OLA (t(76) = 3.02, *p* = 0.034, 95% CI [0.02, 0.88]), with no significant differences relative to DWA or HPA. Neither the θ/β ratio nor the (α + θ)/β ratio yielded significant pairwise differences across scenarios.

### 3.5. Cluster Analysis of α/β Ratio Based on Significant Scenario-Level Differences

Given that AFA produced significantly higher α/β ratios than SRA and OLA in post hoc analyses, we conducted exploratory clustering to identify potential neurophysiological subtypes among participants. K-means clustering yielded optimal separation at K = 2, as determined by elbow-method and silhouette analyses (Figure 5A; Table S3). Hierarchical cluster analysis (HCA) using Euclidean distance and Ward’s linkage method corroborated the K-means solution, producing identical group assignments (Figure 5B). Group I comprised five participants (S01, S10, S13, S15, S17), while Group II included the remaining fifteen participants (S02, S03, S04, S05, S06, S07, S08, S09, S11, S12, S14, S16, S18, S19, S20). Comparative analyses revealed that only the α/β ratio differed significantly between groups (*p* = 0.0028), with Group I exhibiting markedly elevated values relative to Group II (Figure 5C). None of the nine subjective perceptual ratings showed significant between-group differences. These findings suggest that the α/β ratio may serve as a neurophysiological marker distinguishing individual response patterns among visually impaired participants, potentially reflecting trait-like differences in baseline cortical arousal or relaxation states. However, given the small sample size and the exploratory nature of this analysis, these clusters should be regarded as preliminary and hypothesis-generating, requiring validation in a larger, independent sample.

### 3.6. Associations Between EEG Metrics and Subjective Perceptual Ratings

Spearman correlation analyses were conducted to examine relationships between α/β ratios and the nine subjective perceptual metrics across the five scenarios (Figure 6; Tables S4 and S5). Significant positive correlations emerged between α/β ratios and auditory perception metrics within the DWA scenario: sound comfort (*r* = 0.46, *p* = 0.040), sound preference (*r* = 0.52, *p* = 0.019), and sound pleasantness (*r* = 0.50, *p* = 0.026). Within the OLA scenario, α/β ratios correlated positively with sound comfort (*r* = 0.53, *p* = 0.017). In the SRA scenario, α/β ratios showed a significant positive association with environmental preference (*r* = 0.45, *p* = 0.048). Conversely, a significant negative correlation was observed between α/β ratios and odor familiarity in the AFA scenario (*r* = −0.46, *p* = 0.039). This negative correlation should be interpreted with caution, as it may reflect heightened attention or novelty responses to unfamiliar odors rather than relaxation per se. Given the exploratory nature of these analyses, these correlations should be interpreted with caution due to the number of comparisons performed.

To further elucidate these relationships, a linear mixed-effects model (LMM) was fitted with α/β ratio as the dependent variable and four representative subjective ratings—sound preference, odor comfort, environmental comfort, and environmental preference—as fixed-effect predictors, while incorporating participant identity as a random intercept. These four predictors were selected a priori based on their theoretical relevance to the hypothesized relaxation response: sound preference and odor comfort represent domain-specific sensory evaluations, while environmental comfort and environmental preference capture overall environmental appraisal. The remaining subjective ratings were excluded to maintain model parsimony given the sample size (*n* = 20). The model intercept was estimated at β = 2.285 (SE = 0.251, t = 9.11, *p* < 0.001, 95% CI [1.793, 2.776]). The random-intercept variance was σ^2^ = 1.18, with an intraclass correlation coefficient (ICC) of 0.82, indicating that 82% of the total variance in α/β ratios was attributable to between-participant differences (Table 2). However, none of the subjective perceptual predictors reached statistical significance: environmental comfort (β = −0.021, SE = 0.096, *t* = −0.22, *p* = 0.826, 95% CI [−0.208, 0.166]), environmental preference (β = −0.100, SE = 0.103, t = −0.97, *p* = 0.334, 95% CI [−0.302, 0.102]), sound preference (β = 0.002, SE = 0.070, *t* = 0.03, *p* = 0.975, 95% CI [−0.136, 0.140]), and odor comfort (β = 0.046, SE = 0.085, *t* = 0.54, *p* = 0.589, 95% CI [−0.120, 0.211]). The 95% confidence intervals for all predictors included zero, indicating no meaningful linear association between subjective ratings and neurophysiological responses. These results suggest that EEG-derived α/β ratios and subjective perceptual ratings may capture partially independent dimensions of multisensory perception. While subjective ratings reflect conscious evaluative judgments, the α/β ratio may index scene-driven physiological relaxation states that do not covary linearly with explicit comfort or preference judgments. However, this dissociation should be interpreted as a statistical observation within the current experimental context rather than evidence for the independent functioning of subjective and neurophysiological systems.

To examine whether heterogeneity in visual deprivation onset influenced the observed EEG effects, we conducted an additional linear mixed-effects analysis with occipital α/β ratio as the dependent variable, scenario and visual-history group (congenital, *n* = 14; acquired, *n* = 6) as fixed effects, and participant identity as a random intercept. Neither the main effect of group (F = 0.004, *p* = 0.951) nor the scenario × group interaction (F = 0.429, *p* = 0.788) was significant, indicating that the two groups showed highly overlapping α/β distributions across all five scenarios (Figure S1).

## 4. Discussion

### 4.1. Dissociation Between Explicit Appraisal and Implicit Neural Relaxation

A central finding of this study is the dissociation between conscious hedonic appraisal and neurophysiological relaxation indexed by occipital α/β ratios. The Dynamic Water Feature Area (DWA) dominated subjective preference ratings across auditory and environmental dimensions yet did not yield a correspondingly superior α/β ratio relative to the Shaded Rest Area (SRA) or Open Lawn Area (OLA). By contrast, the Aromatic Flowerbed Area (AFA) elicited the numerically highest α/β ratios—statistically indistinguishable from DWA and the Hard-paved Activity Plaza Area (HPA), but significantly exceeding those of SRA and OLA—despite middling subjective ratings. This pattern indicates that DWA’s subjective primacy derives largely from the acoustic salience of flowing water, whereas AFA induced a robust, implicitly expressed relaxation response under a low-amplitude acoustic backdrop that was not proportionately reflected in explicit evaluative judgments.

This dissociation aligns with dual-process frameworks of emotion, wherein explicit evaluative judgments and implicit physiological states are subserved by partially dissociable neural circuits. Subjective preference ratings engage prefrontal evaluative networks shaped by cultural schemas and prior experience, whereas occipital α/β ratios index a more automatic dimension of cortical state modulation, plausibly reflecting attenuated bottom-up sensory processing and reduced allocentric attentional demands. Notably, in visually impaired individuals, occipital α/β activity may also reflect attentional engagement, cognitive load, or cross-modal recruitment of visual cortical areas. Nevertheless, the scenario-specific pattern of α/β elevations and its independence from subjective ratings support its use as a relaxation index in this context, although high-density EEG studies are needed to further disambiguate these interpretations. In the DWA scenario, the dynamic spectrotemporal structure and moderate intensity of water sounds confer acoustic salience that dominates conscious appraisal—a pattern consistent with the privileged role of auditory cues in environmental appraisal in this cohort; whether this contribution is specific to visually impaired populations or reflects a general property of auditory salience cannot be determined without a sighted comparison group. Conversely, AFA elicited robust α/β elevation despite middling subjective ratings. This apparent disconnect may reflect differential recruitment of olfactory–limbic pathways: under low-amplitude acoustic conditions, floral volatiles may engage piriform and insular cortices via thalamic and extrarhinal routes that bypass higher-order evaluative processing, triggering parasympathetic-mediated cortical relaxation without commensurate conscious hedonic amplification. Prior work supports this interpretation: Alvarsson et al. [30] documented decoupled autonomic and cortical responses to natural soundscapes, and animal work suggests that inhaled floral volatiles can engage GABAergic signaling via the olfactory system to produce relaxation-like effects without requiring systemic absorption [31]. The present findings extend these observations to a visually impaired cohort, although it should be noted that this dissociation represents a statistical observation within the current experimental context rather than evidence for the independent functioning of subjective and neurophysiological systems.

### 4.2. Olfactory Novelty and Cortical Relaxation: A Habituation-Based Account

Among the three olfactory perceptual dimensions, only odor familiarity varied significantly across scenarios. The Hard-paved Activity Plaza Area (HPA) elicited the highest familiarity ratings—significantly exceeding those of AFA—while odor comfort and odor preference showed no between-scenario differences. This pattern reflects intuitive everyday experience: HPA’s odor profile (bread, coffee, grilled sausage) represents routine urban olfactory exposures, whereas AFA’s floral bouquet, though potentially pleasant, constitutes a non-routine olfactory event for most participants.

The inverse relationship between odor familiarity and α/β elevation observed in the AFA scenario invites mechanistic interpretation. However, given that familiarity was rated rather than manipulated and that the AFA scenario differed from others in both odor and sound, this account remains tentative pending future factorial designs that independently manipulate odor identity, familiarity, and acoustic context. One parsimonious account invokes olfactory habituation: repeated exposure to familiar urban odors (e.g., bread, coffee) in daily life may diminish their capacity to recruit attentional and autonomic resources upon re-encounter, resulting in attenuated neurophysiological modulation. Conversely, unfamiliar floral scents may initially engage novelty-detection circuits—including the orbitofrontal cortex and amygdala—triggering an orienting response followed by deeper parasympathetic-mediated relaxation once stimulus appraisal concludes. This biphasic pattern would manifest as elevated α/β ratios despite—or precisely because of—low initial familiarity. Alternatively, the elevated α/β response to unfamiliar floral odors may instead reflect heightened attention or novelty-driven arousal rather than relaxation per se, particularly given the relatively short odor presentation duration in the present study, which precludes definitive dissociation of novelty effects from genuine relaxation responses. Such a mechanism finds indirect support in studies of olfactory adaptation, wherein novel odorants produce more sustained activation of piriform and anterior cingulate cortices compared to familiar counterparts. The present findings qualify conventional restorative-environment frameworks, which typically posit familiarity as a facilitator of comfort and relaxation. Instead, under brief exposure conditions, novelty in the olfactory domain may paradoxically enhance physiological relaxation—a pattern that warrants investigation in visually impaired populations, for whom olfactory exploration may assume compensatory significance. This interpretation remains tentative, as the relative contributions of habituation, novelty-driven arousal, and relaxation cannot be fully separated on the basis of the current data. This interpretation converges with Hedblom et al. [32], who reported dissociations between physiological stress markers and consciously reported pleasantness across sensory modalities, while extending the observation to the present visually impaired sample, for whom the generality of this pattern relative to sighted individuals remains to be established.

### 4.3. Cross-Modal Plasticity as a Source of Neurophysiological Heterogeneity

K-means clustering (K = 2, validated by congruent hierarchical cluster analysis) partitioned participants into a high-α/β group (*n* = 5) and a low-α/β group (*n* = 15). Between-group comparisons revealed a significant difference solely in α/β ratios (*p* = 0.0028), with none of the nine subjective perceptual ratings reaching statistical significance. This dissociation between neurophysiological and subjective indices constitutes the empirical cornerstone of the present interpretation.

Crucially, the between-group divergence in baseline α/β ratios persisted across all five scenarios rather than manifesting as a scenario-selective effect. This stability suggests that neurophysiological relaxation capacity constitutes a trait-like characteristic—reflecting persistent neuroarchitectural organization—rather than a transient, state-dependent response to specific sensory cues. Given the small sample size, particularly the high-α/β subgroup (*n* = 5), these clusters should be regarded as exploratory and hypothesis-generating. This inference resonates with extensive evidence for cross-modal reorganization in the visual cortex following early or congenital visual deprivation [33,34,35]. The α/β clustering observed herein may therefore represent an electrophysiological manifestation of individual variability in the extent and functional efficacy of such reorganization, potentially modulated by factors such as age at onset, duration of deprivation, or etiological subtype—variables not systematically captured in the present cohort but that warrant investigation as candidate moderators of neuroplastic outcomes.

This neurophysiological heterogeneity has been systematically underappreciated in both research and clinical practice. Morelli et al. [36] illustrated, via a single-case multisensory rehabilitation program, that tailoring activities to a child’s visual potential and developmental profile can be more appropriate than uniform protocols. Cappagli et al. [37] subsequently demonstrated that individualized, early-initiated audiomotor training produces measurable improvements in spatial cognition and mobility in visually impaired children—empirical evidence that tailored multisensory interventions can harness neuroplasticity when individual profiles are properly characterized. The α/β clustering identified in the present study provides a putative neurophysiological substrate for such individualized approaches. Specifically, the high-α/β subgroup may represent individuals with heightened sensitivity to low-dose olfactory or multimodal stimulation, for whom subtle sensory cues suffice to elicit relaxation responses. Conversely, the low-α/β subgroup may require more intensive sensory modulation to achieve comparable neurophysiological effects. From this perspective, α/β ratios may hold promise not merely as descriptive indices, but as predictive biomarkers of treatment responsiveness in multisensory-based neurorehabilitation protocols.

Linear mixed-effects modeling corroborated this trait-like interpretation, revealing that inter-individual baseline differences—rather than linear coupling with subjective appraisals—drove α/β variance. Spearman correlations further indicated that significant couplings between α/β and subjective metrics were scenario-specific, defying reduction to a single global slope. Collectively, these findings challenge the assumption of neurophysiological uniformity within visually impaired populations and motivate future work incorporating detailed ophthalmologic and neurodevelopmental histories as core covariates.

### 4.4. Methodological Considerations and Future Directions

The controlled auditory–olfactory simulation paradigm adopted herein was designed to examine combined auditory–olfactory environmental experiences under standardized laboratory conditions. Each scenario bundled one soundscape with one odor, without auditory-only, odor-only, or crossed conditions; therefore, the observed effects cannot be unambiguously attributed to a single modality or their interaction. In naturalistic outdoor settings, sensory channels are intrinsically confounded, precluding unambiguous attribution of neurophysiological effects to individual modalities. Within these constraints, the present design achieved contrasts among AFA (floral scent + low-amplitude sound), DWA (water sound), SRA (low-amplitude sound), OLA, and HPA, thereby enabling the detection of AFA’s superior α/β elevation relative to SRA and OLA. This experimental strategy aligns with Hedblom et al.’s [32] use of virtual multisensory environments to approximate real-world sensory combinations under controlled conditions and represents a tractable approach for dissecting the neurobiological substrates of multisensory integration.

Critically, the neurophysiological indices obtained under such controlled conditions retain meaningful ecological validity. Lu and Lau [38] assessed the ecological validity of audiovisual environmental simulations and reported that EEG-derived metrics maintained acceptable correspondence with field-based measurements, even as psychological restoration measures diverged from in situ responses. This dissociation supports the interpretation that the α/β effects observed herein reflect genuine neurophysiological phenomena—phenomena that may be more robust to contextual variation than conscious subjective report.

Building on these methodological foundations, future investigations should extend this line of inquiry along several dimensions. First, systematic stratification by etiology of visual impairment, age at onset, and duration of blindness is essential to delineate the neuroplastic moderators of individual variability. Second, triangulation with complementary physiological modalities—such as heart-rate variability, electrodermal activity, and functional near-infrared spectroscopy—would clarify whether α/β ratios primarily index attentional, autonomic, or affective dimensions of the relaxation response. Third, a phased research trajectory is warranted, progressing from tightly controlled laboratory paradigms, which afford mechanistic specificity, to semi-naturalistic and ultimately in situ deployments that restore ecological complexity. Finally, the identification of α/β as a stable individual-difference marker invites investigation into its predictive utility as a biomarker for treatment response in multisensory-based neurorehabilitation protocols for visually impaired populations. Whether the present findings extend to sighted populations remains unclear, and comparative studies using the same paradigm would help clarify this question.

### 4.5. Limitations

Several limitations warrant acknowledgment. First, the modest sample size (*N* = 20) constrained statistical power, particularly for the high-α/β subgroup (*n* = 5), limiting the stability of the clustering solution. Replication in independent, adequately powered cohorts is therefore essential. An a priori power analysis was not conducted, and the present sample size reflects the practical constraints of recruiting from this specific population. Second, the study did not systematically stratify participants by ophthalmologic and neurodevelopmental variables—including etiology, age at onset, and duration of blindness—which prior work identifies as critical moderators of cross-modal reorganization [35]. Detailed clinical profiling (e.g., residual vision, medication use, hearing and olfactory status, and neurological history) was not systematically collected, and these variables should constitute core covariates in future expanded-sample designs. Moreover, the absence of a sighted comparison group precludes attribution of the observed effects to visual-impairment-specific mechanisms; comparative studies with sighted cohorts are needed to distinguish impairment-specific responses from general multisensory effects. It is therefore unknown whether the observed patterns—particularly the dissociation between subjective appraisal and α/β relaxation—are unique to visual impairment or would also emerge in sighted individuals. Third, EEG analysis was confined to three spectral ratios (α/β, θ/β, (α + θ)/β); while parsimonious, this approach captures only a subset of relevant neurophysiological dynamics. Fourth, participants were tested alone in the simulated environments, which may not fully reflect the social context of real-world mobility. Fifth, only cortical EEG activity was measured; autonomic signals were not recorded. Finally, the laboratory-based simulation necessarily attenuated ecological complexity. Real-world environments present concurrent non-audiovisual cues—including airflow, temperature, humidity, turbulence, concentration gradients, tactile feedback from ground surfaces, and social contact—that cannot be fully replicated in a laboratory setting, as well as extended dwell times that were reduced to brief, controlled exposures. The present findings therefore reflect acute responses to brief (2 min) exposures rather than evidence of sustained relaxation or long-term benefits; repeated exposure sessions would be needed to determine whether α/β responses remain stable or diminish with increasing stimulus familiarity. Similarly, although the olfactory delivery system maintained a constant flow rate (0.1 mL/s) and included inter-stimulus ventilation to minimize carryover, absolute odor concentration in the breathing zone was not directly measured, and the 5 min ventilation protocol was designed to minimize residual odorants but was not verified by instrumental measurement. Formal audiometric and olfactory threshold testing were also not conducted. Accordingly, extrapolations to authentic outdoor settings should be conservative pending field-based validation.

## 5. Conclusions

This study provides preliminary evidence of a dissociation between explicit hedonic appraisal and implicit neurophysiological relaxation in visually impaired individuals exposed to naturalistic auditory–olfactory environments. Elevated occipital α/β ratios in response to floral olfactory cues embedded within low-amplitude acoustic contexts—despite middling subjective preference—suggest that cortical relaxation and conscious evaluative judgment may follow partially independent processing trajectories. The inverse relationship between odor familiarity and α/β elevation challenges conventional assumptions that familiar sensory environments are inherently more restorative. However, this finding may also reflect novelty-driven attentional responses rather than relaxation per se. Critically, clustering analysis revealed potentially stable individual differences in neurophysiological baselines that were not captured by subjective ratings, suggesting neurophysiological heterogeneity within the visually impaired population that may reflect individual variability in cross-modal reorganization. Given the exploratory nature of these analyses, the modest sample size, the restricted age range, the heterogeneity of visual impairments, and the acute experimental paradigm, these findings should be regarded as hypothesis-generating rather than firm evidence. While the controlled laboratory paradigm limits ecological generalizability, this work provides initial neurophysiological evidence that multisensory landscape elements can modulate cortical state through mechanisms that appear partly dissociated from conscious preference. Future studies should employ larger, better-characterized samples with repeated exposure sessions and high-density EEG to validate these preliminary patterns and delineate the boundary conditions of multisensory relaxation effects in visually impaired populations.

## Figures and Tables

**Figure 1:**
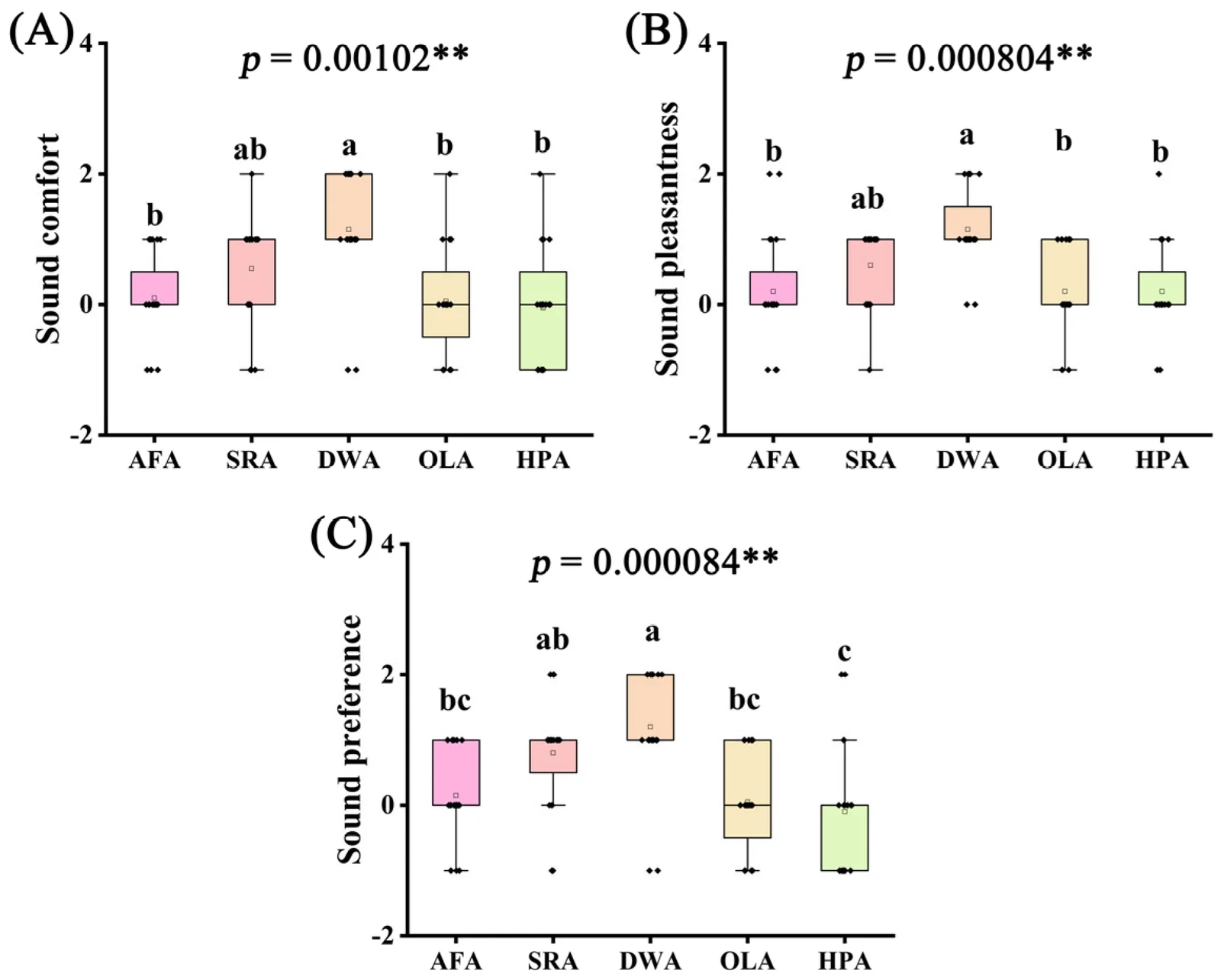
Comparison of three auditory perception metrics across the five simulated scenarios. (**A**) Sound comfort; (**B**) sound pleasantness; (**C**) sound preference. Different lowercase letters denote statistically significant differences between scenarios at *p* < 0.05. “**” indicates *p* < 0.01.

**Figure 2:**
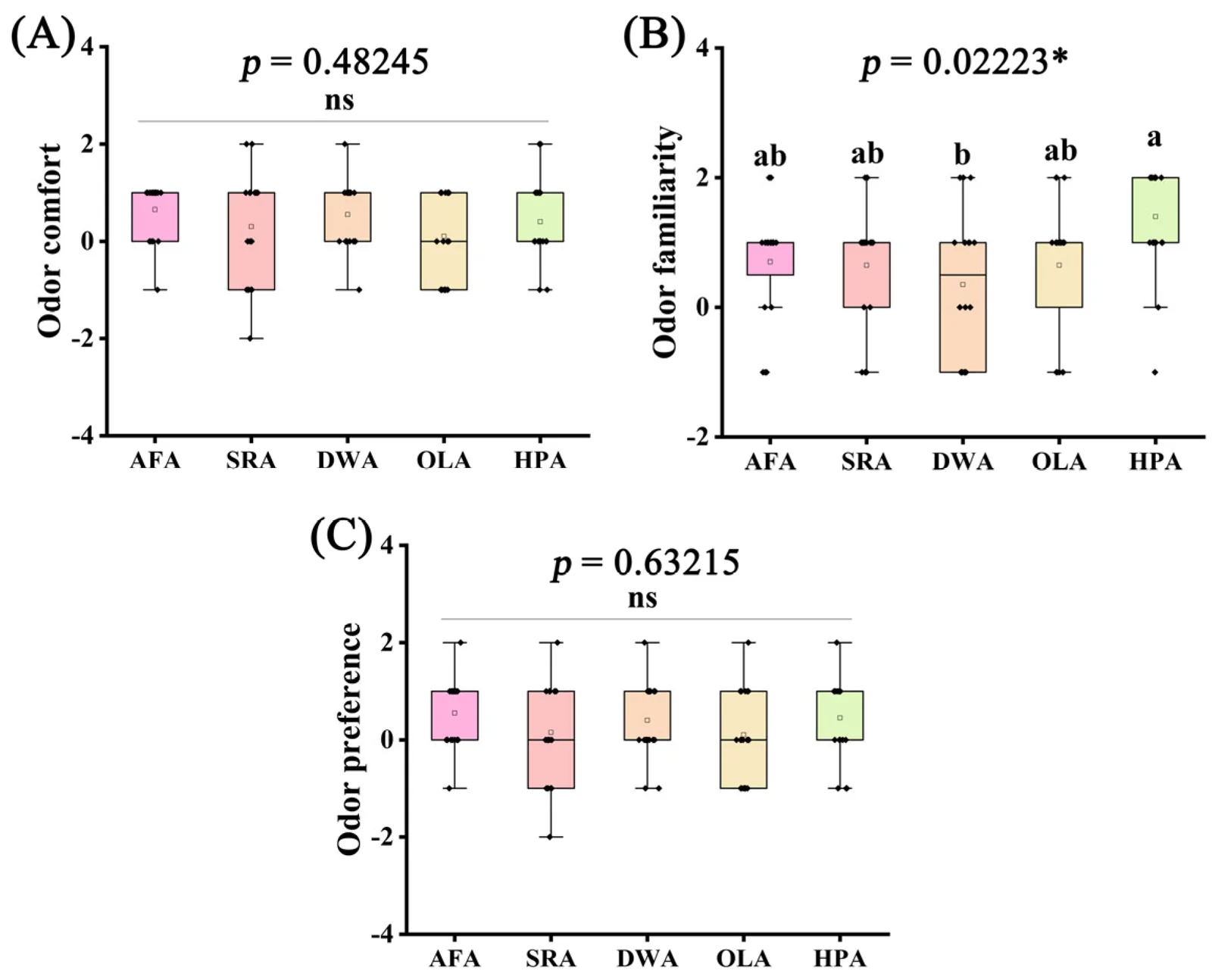
Comparison of three olfactory perception metrics across the five simulated scenarios. (**A**) Odor comfort; (**B**) odor familiarity; (**C**) odor preference. Different lowercase letters denote statistically significant differences at *p* < 0.05. “*” indicates *p* < 0.05. “ns” indicates *p* > 0.05.

**Figure 3:**
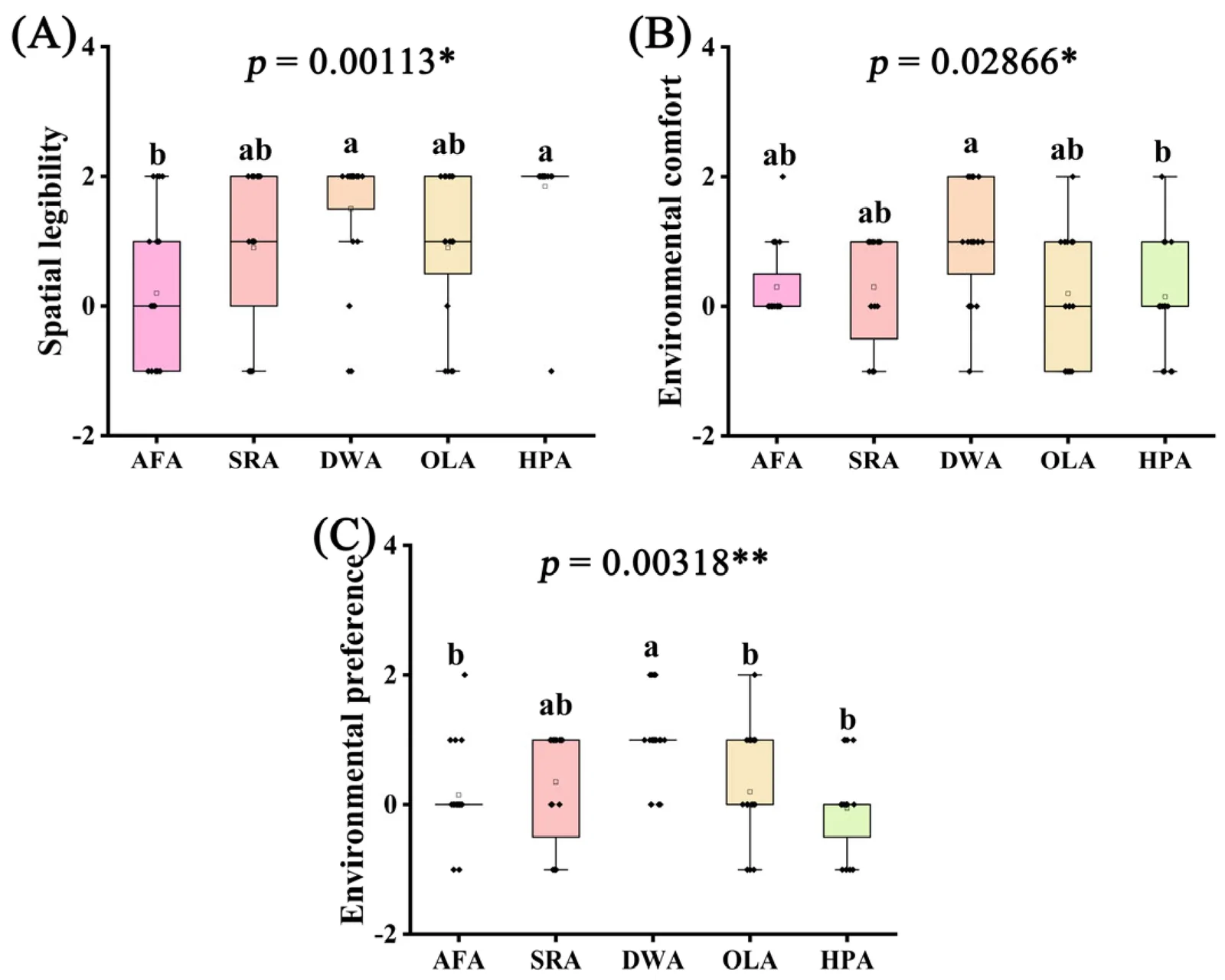
Comparison of three integrated auditory–olfactory perception metrics across the five simulated scenarios. (**A**) Spatial legibility; (**B**) Environmental comfort; (**C**) Environmental Preference. Different lowercase letters denote statistically significant differences at *p* < 0.05. “*” indicates *p* < 0.05. “**” indicates *p* < 0.01.

**Figure 4:**
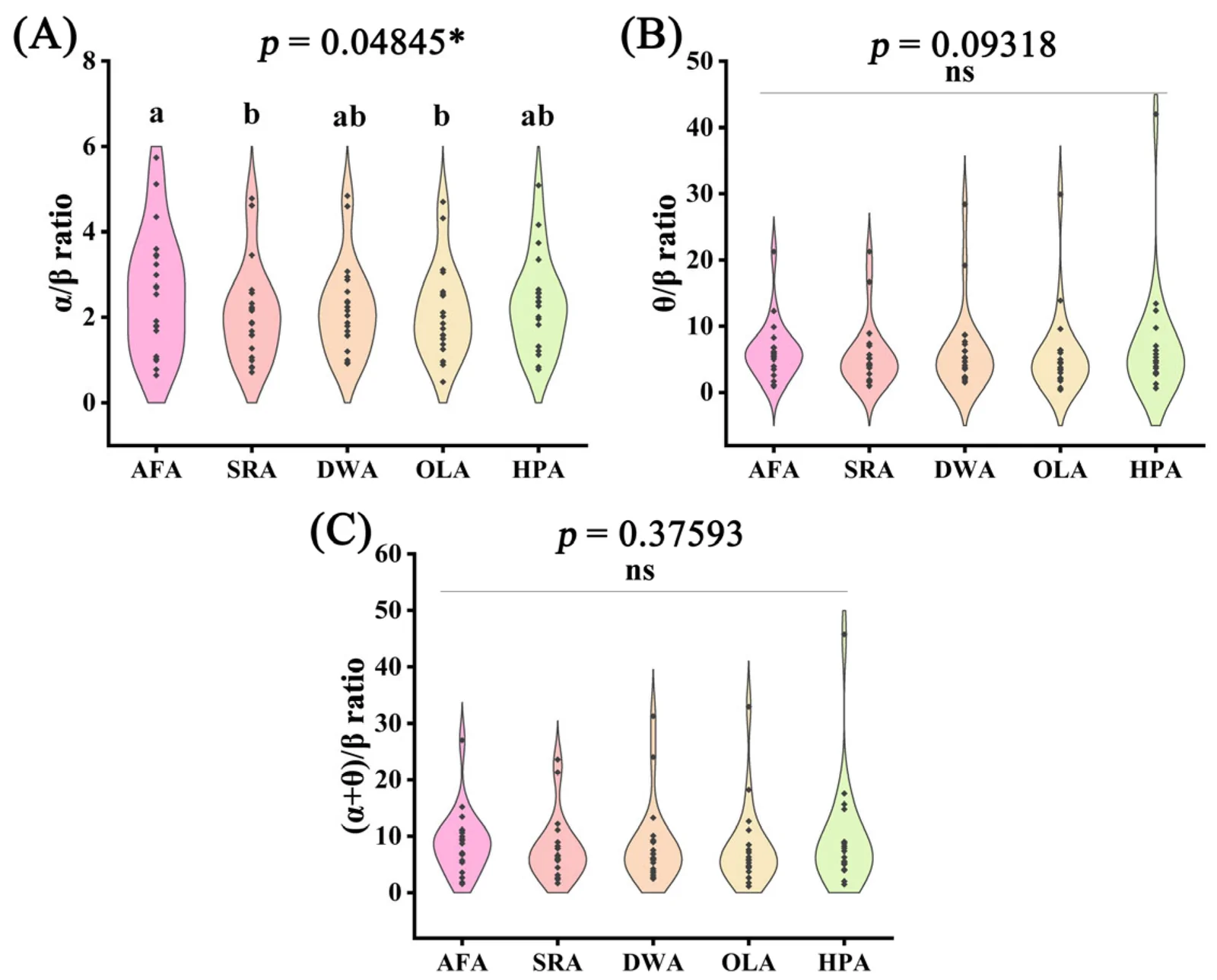
Comparison of three EEG-derived metrics across the five simulated scenarios. (**A**) α/β ratio; (**B**) θ/β ratio; (**C**) (α + θ)/β ratio. Different lowercase letters denote statistically significant differences at *p* < 0.05. “*” indicates *p* < 0.05. “ns” indicates *p* > 0.05.

**Figure 5:**
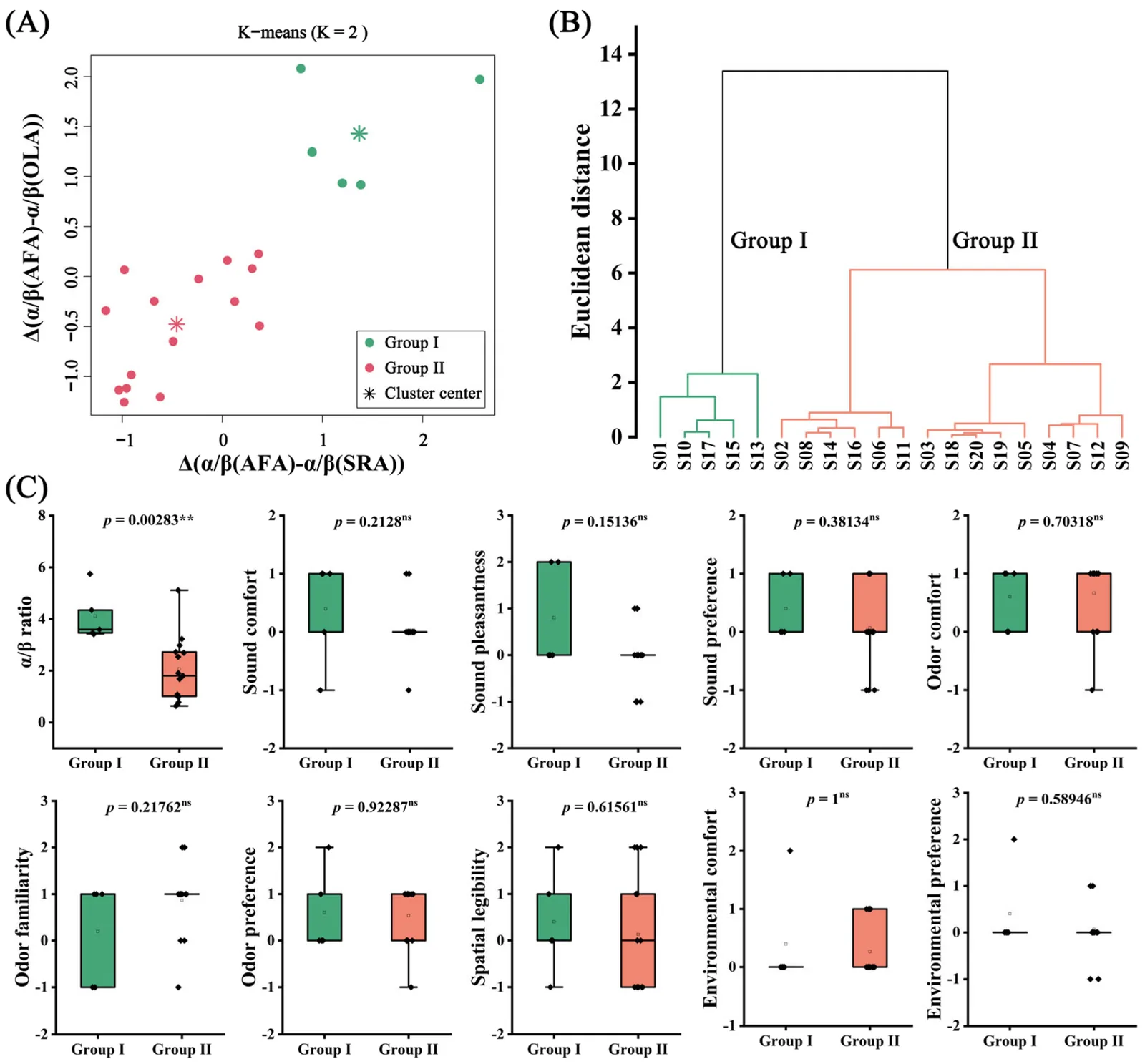
Cluster analysis of α/β ratios based on scenario-level differences between AFA versus SRA and OLA. (**A**) K-means clustering (K = 2); (**B**) hierarchical cluster analysis using Ward’s method; (**C**) between-group comparisons of α/β ratios and subjective perceptual ratings. “**” indicates *p* < 0.01. “ns” indicates *p* > 0.05.

**Figure 6:**
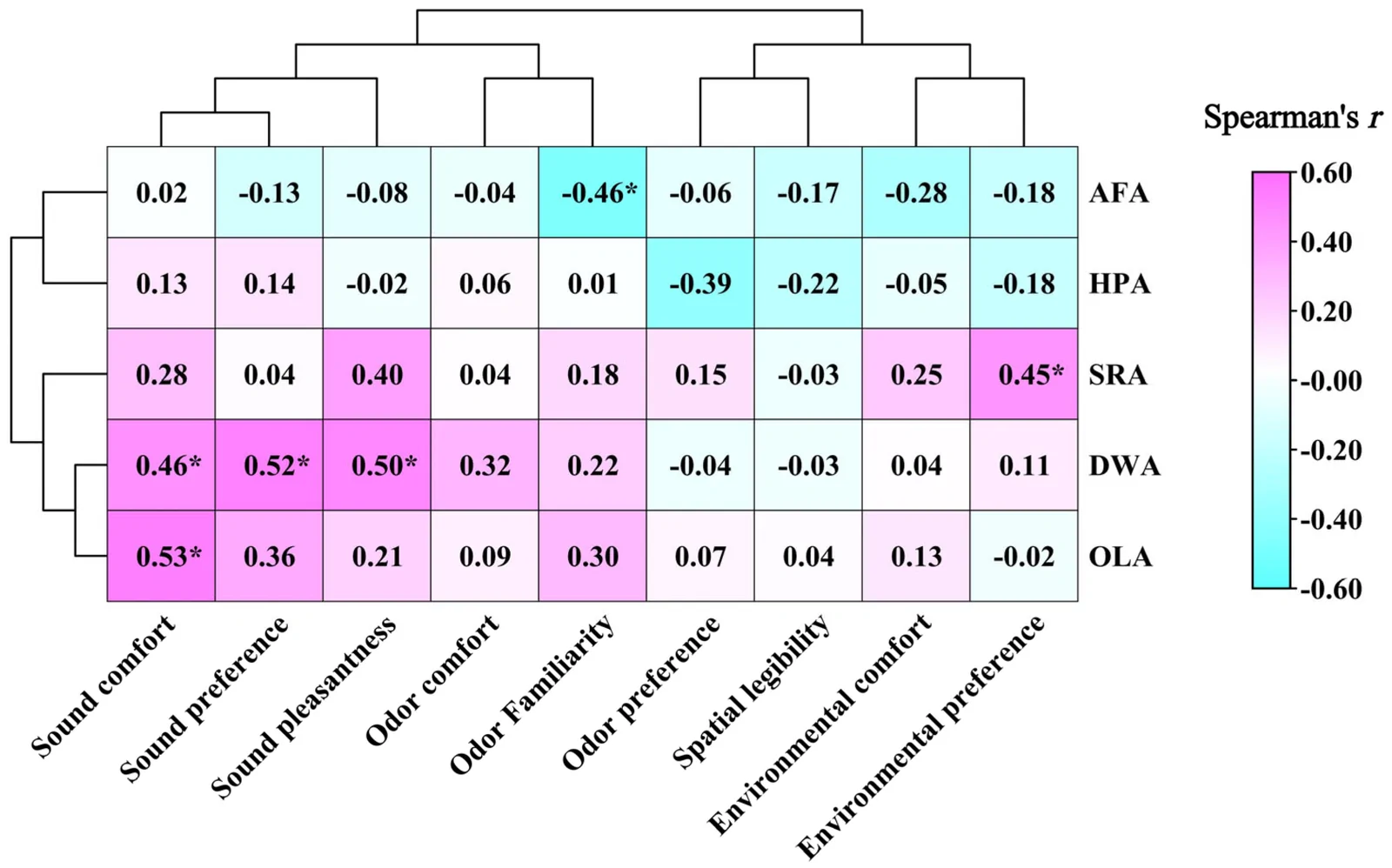
Clustered Spearman correlation heatmap of α/β ratios and subjective perceptual metrics across five simulated scenarios. “*” indicates *p* < 0.05.

**Table 1:** Summary of experimental scenario characteristics.

No.	Scenario Type	Scenario Description	Auditory Elements	Olfactory Elements
1	Aromatic Flowerbed Area (AFA)	Dedicated horticultural area featuring densely arranged aromatic flowering species. The design prioritizes olfactory experience through concentrated floral displays that attract pollinators and visitors alike.	Buzzing of bees, faint rustling of petals in the breeze, and birdsong. The soundscape is dominated by biotic and natural sounds at relatively low sound pressure levels (approx. 40–50 dB). Field recordings were obtained from Rose Manor, Gaoyao District, Zhaoqing, China.	Osmanthus, jasmine
2	Shaded Rest Area (SRA)	Shaded recreational zones characterized by dense canopy cover and integrated seating, providing opportunities for relaxation and informal social interaction. The spatial configuration emphasizes natural shelter and thermal comfort.	Birdsong, rustling of wind through foliage, and faint distant human voices. The soundscape comprises predominantly natural acoustic elements at low sound pressure levels (approx. 40–50 dB). Field recordings were obtained from Seven Star Crags (Qixingyan) Scenic Area, Duanzhou District, Zhaoqing, China.	Forest floor/woodland, camphor tree
3	Dynamic Water Feature Area (DWA)	Water-centered landscape zones incorporating moving water elements such as fountains, streams, or cascades. These features provide dynamic visual and auditory stimuli that enliven the overall landscape atmosphere.	Flowing water (e.g., babbling brooks, cascading flows) accompanied by intermittent birdsong. The soundscape is water-dominated at moderate sound pressure levels (approx. 50–60 dB). Field recordings were obtained from Ziyun Valley, Gaoyao District, Zhaoqing, China.	Water vapor/aquatic freshness, lotus, reed
4	Open Lawn Area (OLA)	Expansive turfgrass areas accommodating informal recreation such as picnicking and leisure activities. The spatial character emphasizes openness, unobstructed views, and exposure to natural ambient conditions.	Rustling of grass in the wind, distant children’s play sounds, and birdsong. The soundscape combines aeolian and anthropogenic sounds at variable sound pressure levels (approx. 45–55 dB). Field recordings were obtained from Paifang Park, Duanzhou District, Zhaoqing, China.	Fresh-cut grass, moist earth/soil
5	Hard-paved Activity Plaza Area (HPA)	Hardscaped open squares surfaced with stone or concrete, designed to accommodate collective activities, exercise, and public gatherings. The built environment is characterized by prominent artificial elements and functional programming.	Footsteps, conversational hubbub, amplified music, activity-related sounds, and vehicular noise. The soundscape is dominated by anthropogenic sounds at relatively high sound pressure levels (approx. 60–70 dB). Field recordings were obtained from Yanqian Village, Duanzhou District, Zhaoqing, China.	Bread, coffee, grilled sausage

**Table 2:** Linear mixed-effects model predicting α/β ratio from subjective ratings.

Predictor	Estimate (β)	Std. Error	df	*t* Value	*p* Value	95% CI
(Intercept)	2.285	0.251	19.676	9.110	1.71 × 10^−8^ ***	[1.793, 2.776]
Environmental comfort	–0.0211	0.0955	77.0715	–0.221	0.826	[–0.208, 0.166]
Environmental preference	–0.1004	0.103	78.624	–0.972	0.334	[–0.302, 0.102]
Sound preference	0.00217	0.0703	78.230	0.031	0.975	[–0.136, 0.140]
Odor comfort	0.045878	0.0845	79.568	0.543	0.589	[–0.120, 0.211]
**Random Effects**	**Estimate (β)**	**Std. Error**	**df**	***t* Value**	***p* Value**	**95% CI**
σ^2^_participant	1.183	-	-	-	-	-
σ^2^_residual	0.252	-	-	-	-	-
ICC	0.824	-	-	-	-	-

Note: *** indicates *p* < 0.001.

## Data Availability

The data presented in this study are available on reasonable request from the corresponding author. The data are not publicly available due to privacy and ethical restrictions imposed by the institutional review board.

## Supplementary Materials

The following supporting information can be downloaded at https://www.mdpi.com/article/10.3390/biology15191705/s1. Figure S1: Occipital α/β ratios by scenario and visual-history group. Congenital (*n* = 14) and acquired (*n* = 6) groups showed highly overlapping distributions across all five scenarios. LMM: group main effect, F(1, 18) = 0.004, *p* = 0.951; scenario × group interaction, F(4, 72) = 0.429, *p* = 0.788; Table S1: Participant demographic and clinical characteristics. Table S2: Subjective rating scale for perceptual evaluation; Table S3: Determining the optimal K in K-means clustering via the Silhouette Coefficient Method; Table S4: Spearman correlation matrix between α/β ratios and nine subjective perceptual metrics across the five simulated scenarios; Table S5: Matrix of *p*-values for Spearman correlations between α/β ratios and nine subjective perceptual metrics across five simulated scenarios. Audio S1: Auditory stimuli used in the five simulated environmental scenarios (AFA, SRA, DWA, OLA, HPA).
