# Supplementary material for: Auditory–Olfactory Integration in Simulated Natural Environments Dissociates Neural and Subjective Responses in Visually Impaired Individuals
Source: Biology (Basel). 2026 Sep 24;15(19):1705. doi: 10.3390/biology15191705 (PMC13663358; doi:10.3390/biology15191705)
Supplement: Supplementary file 1 [file biology-15-01705-s001.zip › Figure S1.pdf]

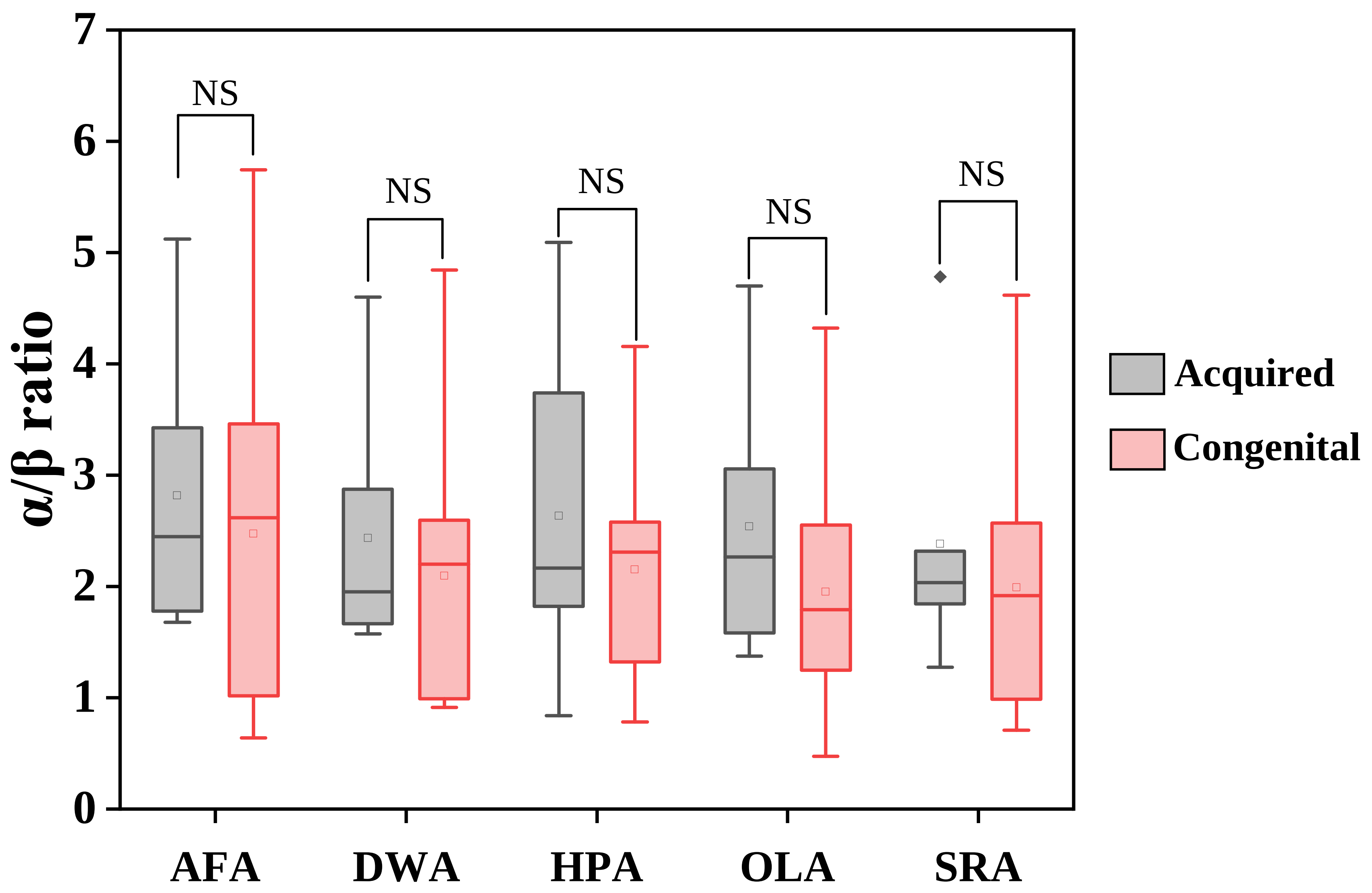
α/β ratio
0
1
2
3
4
5
6
7
NS
NS
NS
NS
NS
AFA
DWA
HPA
OLA
SRA
Acquired
Congenital
